# Syk Inhibitors: New Computational Insights into Their Intraerythrocytic Action in *Plasmodium falciparum* Malaria

**DOI:** 10.3390/ijms21197009

**Published:** 2020-09-23

**Authors:** Giuseppe Marchetti, Alessandro Dessì, Roberto Dallocchio, Ioannis Tsamesidis, Maria Carmina Pau, Francesco Michelangelo Turrini, Antonella Pantaleo

**Affiliations:** 1 Department of Biomedical Sciences, University of Sassari, 07100 Sassari, Italy; johntsame@gmail.com (I.T.); paumc81@tiscali.it (M.C.P.); apantaleo@uniss.it (A.P.); 2 National Research Council (CNR) Institute of Biomolecular Chemistry, 07100 Sassari, Italy; roberto.dallocchio@cnr.it; 3 UMR 152 Pharma-Dev, Université de Toulouse III, IRD, UPS, 31000 Toulouse, France; 4 Department of Oncology, University of Turin, 10126 Turin, Italy; francesco.turrini@unito.it

**Keywords:** band 3, red blood cells, antimalarial drugs, molecular docking, molecular dynamics

## Abstract

Resistance to antimalarial drugs has spread rapidly over the past few decades. The WHO recommends artemisinin-based combination therapies for the treatment of uncomplicated malaria, but unfortunately these approaches are losing their efficacy in large areas of Southeast Asia. In 2016, artemisinin resistance was confirmed in 5 countries of the Greater Mekong subregion. We focused our study on Syk inhibitors as antimalarial drugs. The Syk protein is present in human erythrocytes, and the membrane of protein band 3 is its major target following activation by oxidant stress. Tyr phosphorylation of band 3 occurs during *P. falciparum* growth, leading to the release of microparticles containing hemicromes and structural weakening of the host cell membrane, simplifying merozoite reinfection. Syk inhibitors block these events by interacting with the Syk protein’s catalytic site. We performed in vitro proteomics and in silico studies and compared the results. In vitro studies were based on treatment of the parasite’s cellular cultures with different concentrations of Syk inhibitors, while proteomics studies were focused on the Tyr phosphorylation of band 3 by Syk protein with the same concentrations of drugs. In silico studies were based on different molecular modeling approaches in order to analyze and optimize the ligand–protein interactions and obtain the highest efficacy in vitro. In the presence of Syk inhibitors, we observed a marked decrease of band 3 Tyr phosphorylation according to the increase of the drug’s concentration. Our studies could be useful for the structural optimization of these compounds and for the design of novel Syk inhibitors in the future.

## 1. Introduction 

Spleen tyrosine kinase (Syk) is a cytosolic non-receptor tyrosine kinase that functions downstream of antigen receptors in immune cells such as mast cells, B lymphocytes, and macrophages. Syk is a crucial signal transducer of activated immunoreceptors in multiple downstream events, which differ depending on the cell type, including proliferation, differentiation, and phagocytosis [1,2,3]. Syk function might, therefore, be an attractive target for therapeutic interventions for autoimmune or inflammation diseases [4]. Syk is composed of two Src homology tandem domains, defined as N-SH2 and C-SH2 [5]; these are important for activity regulation and for localizing this kinase in the cell membrane. The tandem SH2 (tSH2) module is also separated by an inter-SH2 linker of 50 amino acids. This is the most conserved region in the kinase family, with 65% sequence homology. Additionally, tSH2 presents an α-helix, which has an important role in protein–protein interactions and serves as a docking platform for tyrosine-based immune receptor activating motifs (ITAMs), which are displayed on the cytosolic side of the plasma membrane [6,7,8]. An interdomain linker of 80–100 amino acids is located between C-SH2 and the catalytic domain. This interdomain is important in regulating kinase activity because it contains phosphotyrosine residues. A catalytic domain or SH1 containing 300 amino acids follows the interdomain linker. It contains the binding sites for ATP and two autophosphorylation sites (Tyr525 and Tyr526) [9]. Syk protein ends with a C-terminal tail, the function of which is currently unidentified.

**Scheme 1:**
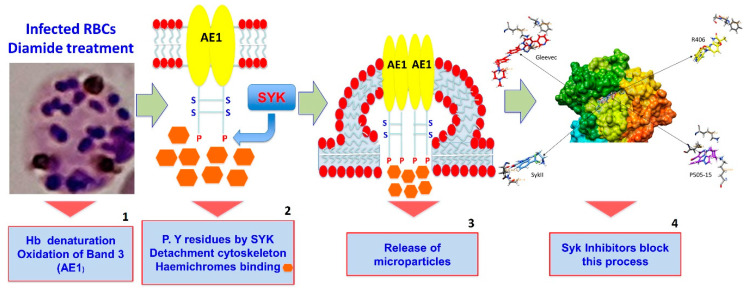
Schematic model of the proposed mechanism of Syk inhibitor action. (**1**) Diamide treatment induces band 3 oxidation and hemoglobin denaturation. (**2**) Syk binds to band 3 and catalyzes band 3 cytoplasmic domain tyrosine phosphorylation, causing its detachment from the cytoskeleton. (**3**) Oxidized and phosphorylated band 3 forms large clusters, which are released in microparticles through the vesiculation. (**4**) Syk inhibitors block this process.

During previous studies of human erythrocyte membranes, we observed that the erythrocytes possess a mechanism that is involved in the expulsion of denatured hemoglobin, requiring the activation of Syk [10,11,12,13,14]. This function could play a role in the process of asexual *P. falciparum* growth, as malaria parasites exert oxidative stress in erythrocytes, causing denaturation of hemoglobin, the oxidation of band 3, and its subsequent phosphorylation by Syk [15,16]. The protein band 3 (also known as the anion exchanger, AE1) constitutes the major attachment site of the spectrin-based cytoskeleton to the erythrocyte’s lipid bilayer, and thereby contributes critically to the stability of the red cell membrane [13,17]. Under steady conditions, this linkage confers to the membrane the required elasticity and mechanical stability. The oxidation of band 3 and its subsequent phosphorylation by Syk kinase cause its detachment from the cytoskeleton and the destabilization of the membrane with the release of microvesicles [10,14]. Scheme 1 shows a schematic model representing the proposed mechanism of action.

Taking in consideration that Syk kinase inhibitors block the expulsion of denatured hemoglobin and its accumulation inside the parasitized erythrocytes [15,16,17], more knowledge of the mechanisms responsible for the protein-ligand recognition and binding will facilitate the design, development, and discovery of a new promising class of antimalarial drugs.

**Figure 1:**
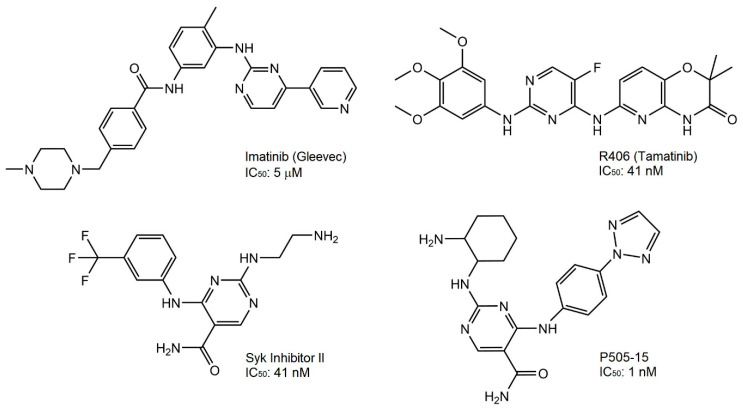
Chemical structures and IC_50s_ for the Syk catalytics subunits of Imatinib, R406, Syk inhibitor II, and P505-15.

We first characterized the ligands with the aim of screening new drugs more efficiently in order to cure malaria, using quantum mechanics (QM) and molecular descriptors to assess the electronic density, molecular electrostatic potential (MEP), and the charge distribution. Molecular modelling approaches, such as docking and molecular dynamics (MD) simulation [18,19], were performed to analyze the binding mode of Syk/ligands. In an effort to pursue a more unbiased approach towards identifying protein tyrosine kinase (PTK) inhibitors with antimalaria activity, we screened some ATP-competitive inhibitors of Syk characterized by different IC_50s_ (Half maximal inhibitory concentrations) for the Syk catalytic subunits [17]. Figure 1 shows the chemical structures and the IC_50s_ values of Imatinib, R406, Syk inhibitor II, and P505-15.

Imatinib, sold under the brand names Gleevec, is a well-tolerated tyrosine kinase inhibitor. It is FDA-approved for use in children [20,21,22] and prevents parasite-induced tyrosine phosphorylation of band 3 and terminates *P. falciparum* parasitemia in vitro by blocking parasite egress at clinically relevant concentrations [15]. This drug is also used for the treatment of chronic myeloid leukemia (CML), acute lymphoblastic leukemia (ALL), and gastrointestinal stromal tumor (GIST). R406 (tamatinib) is an active metabolite of prodrug R788 (fostamatinib), which has already been used in clinical trials for rheumatoid arthritis [23], autoimmune thrombocytopenia [24], autoimmune hemolytic anemia, IgA nephropathy, and lymphoma [25,26].

P505-15 is a candidate drug that has already been used in in vivo studies in mice for treatment of rheumatoid arthritis, non-Hodgkin lymphoma (NHL), and chronic lymphocytic leukemia (CLL) [27,28]. Syk inhibitor II is already used for inhibition of serotonin (5-HT) release in rat basophilic leukemia (RBL) cells and to treat allergic diseases [29,30].

Here, we document the interactions between Syk and its ligands in order to understand the biology at the molecular level, with the aim of improving and modifying the compounds’ structures and discovering new drugs to inhibit infected RBCs.

## 2. Results and Discussion 

### 2.1. Densitometric Analysis of Band 3 Tyrosine Phosphorylation in Diamide-Treated Erythrocytes after Treatment with Syk Inhibitors

**Figure 2:**
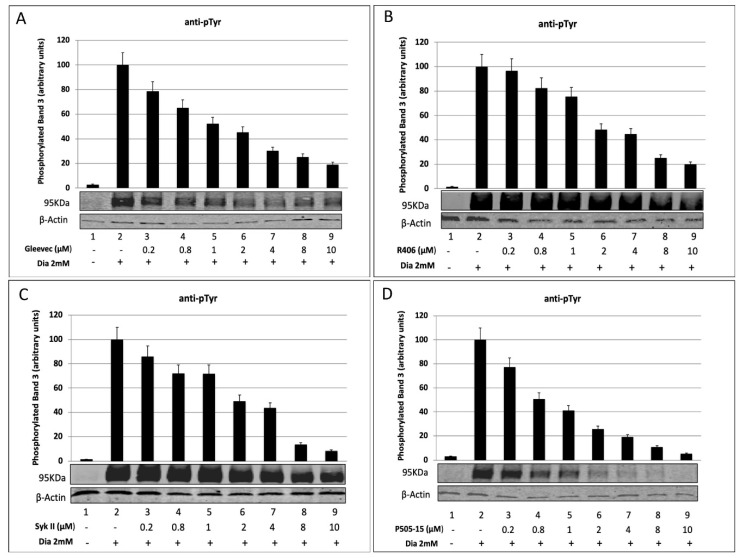
Dose–response course of erythrocyte membrane proteins treated with an oxidant agent and Syk inhibitors related to quantitative analysis of band 3 tyrosine phosphorylation. Erythrocytes were treated with 2 mM diamide (Dia) and different concentrations of the Syk inhibitors (0–10 μM) (**A**) Gleevec, (**B**) R406, (**C**) Syk II, and (**D**) P505-15. **Lane 1** shows the untreateded control sample. Erythrocytes were separated by 8% SDS-PAGE, blotted on a nitrocellulose membrane, and stained with antiphosphotyrosine (apTyr) antibodies. Images were acquired using a laser IR fluorescence detector (Odyssey, Licor, USA). Band 3 Tyr phosphorylation was quantified using Image J software. Values are the means ± for four independent experiments, normalized to total beta-actin levels. All graphs show relative phosphorylation, expressed as a percentage of the maximum observed in each experiment (100%). The error bars represent the standard deviation (SD) of the data.

In vitro studies have shown that diamide treatment increases the band 3 tyrosine phosphorylation state, suggesting a possible functional connection between membrane oxidative damage and modulation of signal transduction pathways involving kinases or phosphatases [31,32]. To better understand the relationship between the activity of Syk inhibitors and their abilities to reduce band 3 tyrosine phosphorylation, we carried out experiments using 2 mM diamide as the control (Figure 2A–D, lane 2) and 2 mM diamide in presence of increasing Syk inhibitor concentration ranging from 0.2 µM to 10 µM (Figure 2A–D, lanes 3–9). As we expected, in accordance with previous reports, diamide caused a rapid band 3 Tyr phosphorylation, without any other phosphorylative changes occurring in erythrocyte membrane proteins [11,33], while Syk inhibitors led to a substantial decrease of band 3 phosphorylation (95–250 KDa) related to the increasing amounts of inhibitors. This trend is clearly evident at 95 KDa bands (phosphorylated band 3).

All densitometry analyses performed on membranes scanned on the Odyssey CLx were done using Odyssey 3.0 software, confirming the large decreases of Tyr phosphorylation levels in band 3 residues caused by Syk protein. Diamide-treated erythrocytes (Figure 2, lane 2) showed high levels of phosphorylation due to the oxidative stress conditions. All the tested Syk inhibitors efficiently suppressed band 3 phosphorylation (Figure 2A–D, lanes 3–9). Table 1 shows the IC_50_ values obtained by densitometric analysis; these results were in agreement with those obtained in previous published studies treating RBCs with different concentrations of each drug and quantitating residual parasitemia 24 and 48 h later (Table 2) [15,17]. P505-15 was the most potent Syk inhibitor, with an IC_50_ of 0.64 μM, while Syk II was the least efficient, with an IC_50_ of 1.72 μM. 

### 2.2. Assessment of Syk Inhibitor Efficacy through Computational Studies

To contribute to the understanding of the mechanism of Syk kinase inhibitors in the treatment of malaria and the possible role of Syk inhibition in parasite growth via suppression of band 3 phosphorylation, we also performed computational studies. This kind of approach has been used by many research groups around the world, e.g., by the “Global Online Fight Against Malaria” project of The Scripps Research Institute (TSRI) in La Jolla, CA, U.S.A. In this study, we used the same techniques and the same software, with the aim of speeding up the knowledge acquisition and the process of antimalarial drug discovery. Antimalarial drugs are as effective as artemisinin derivatives, thus providing new hope for the control of malaria. 

**Figure 3:**
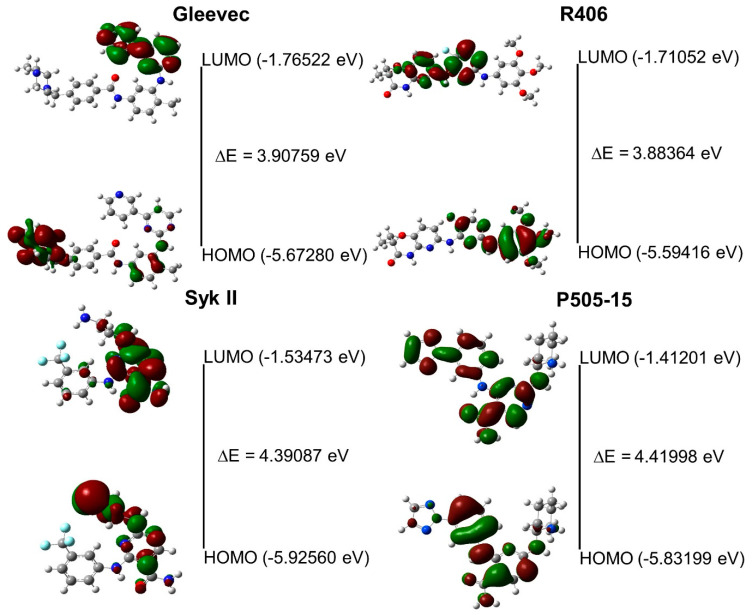
The most probable statistical positions in the highest occupied molecular orbital–lowest unoccupied molecular orbital (HOMO-LUMO) descriptor values.

Previous studies showing the conformational research on R406 [34], Gleevec, Syk inhibitor II, and P505-15 inhibitors [35] were taken into consideration to select the conformation level with the lowest energies and best stability. The highest occupied molecular orbital–lowest unoccupied molecular orbital (HOMO-LUMO) distribution values, energy values, and energy gaps for the hit molecules were computed in order to understand the biological activity [36] (Figure 3).

**Figure 4:**
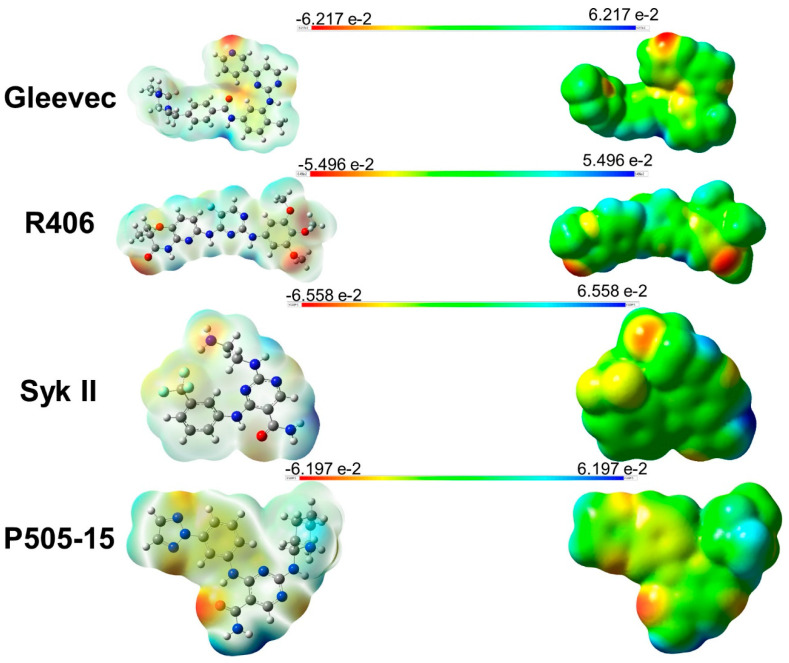
Molecular electrostatic potential (MEP) of surface Syk inhibitors. The color scheme ranges from red (negative potential) via green (zero potential) to blue (positive potential). The unit of isosurface potential is electrostatic potential (eV).

Figure 4 shows the molecular electrostatic potential (MEP) computed using GaussView 5.0, which allowed us to visualize several sites with abundant electrons by analyzing the charge distributions within a molecule in three dimensions. These maps were used to predict how molecules interact with the binding site of Syk.

The X-ray crystal structure for 4FL2.pdb (2.19 resolution) was available in the RCSB PDB database; this structure was the most complete, although the active site compared to other structures found in the same database was forced by an activation loop. 

The docking results obtained from the crystal structure of Syk (4FL2) in the complex with ligands showed conserved H-bond interactions (Table 3); however, a different disposition of ligands in the pocket was observed compared to the poses of the crystal structure of reference.

**Figure 5:**
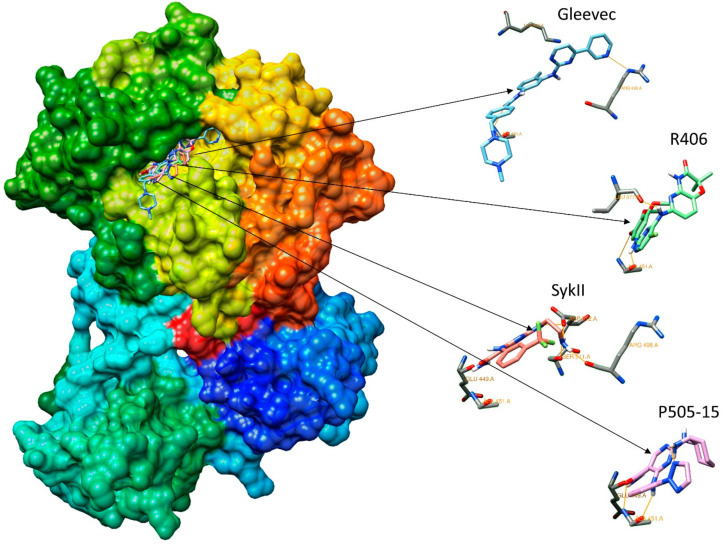
The 3D surface structure of Syk protein, showing Syk inhibitors interacting in the catalytic site. The compounds, with the main H-bond interactions, were reported alone, outside of the pocket of binding.

Although the ligands have a common pattern of interaction with Syk, which is known for all tyrosine kinases [37], our molecules presented different rotations and torsions in the site binding, with the exception of P505-15 [38], which showed an RMSD of 1.39 Å related to its reference (PDB accession number 4RX9), (Figure 5).

Further analysis of the docking demonstrated that the amino acids Leu377, Val385, Ala400, Met448, Met450, Ala451, Gly454, and Leu501 present in the active site of the protein interacted with all tested Syk inhibitors. Table 4 shows an interesting hydrophobic interaction of Tyr525 with Gleevec, representing an autophosphorylation site of Syk protein that was previously found [39]. Gleevec interacts through H-bonds with Lys402, Arg498, and Asp512. R406 shows H-bonds with Leu377 and Ala451. Syk II interacts with Glu449, Ala451, Arg498, Ser511, and Asp512, while P505-15 shows H-bonds with Glu449 and Ala451.

The different interactions between ligands and proteins observed through their docking and different conformations might be due to the fact that the pocket is capped by an activation loop, causing a steric hindrance. Due to the presence of this buried active site, we considered the 4FL2 crystal’s structure in its active conformation compared to the other proteins that we have analyzed in the RCSB database, where their binding pockets were opened. 

The main differences among these structures are in the activation loops (a.a. 520–534) [40], which translate into the active conformation of Syk, closing the ligands in the pocket. The active conformation is characterized for containing the Syk activation loop that closes the ATP-binding pocket once a ligand interacts with the amino acids in the site.

The estimation of the inhibition constants (Ki) of all tested compounds obtained by docking simulation showed that the lowest value was for Gleevec (13.65 nM). This result was unexpected; we assume that it could be due to the increasing number of protein–ligand interactions, which enhance the stability inside the pocket. Table 5 shows the binding energy and Ki values obtained by computational analysis. These data, which were obtained by combining the results of both tests, are useful in understanding the role of Syk inhibitors and their ability to enable the implementation of in vitro testing.

Dynamic molecular analyses were performed to compute the ligands’ trajectory and their interactions with the protein. P505-15 and R406, both alone or in combination with the crystal structure of Syk (4FL2), showed higher chemical stability if compared to Gleevec and Syk II, showing a lower conformation change in the pocket. 

**Figure 6:**
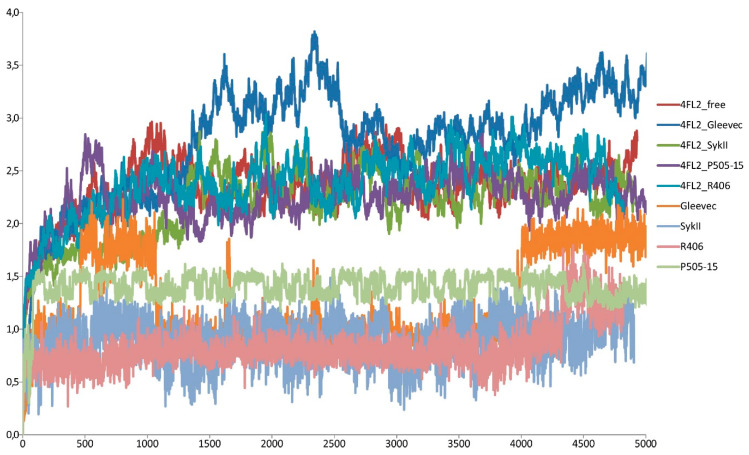
Root mean square deviation (RMSD) values (values on the *y*-axis reported in Å) for all deviation atoms calculated over 10 ns, with the values on the *x*-axis reported as steps (1 step = 2 ps) of 4FL2, the compounds alone, and the four complexes—4FL2-Gleevec, 4FL2-SykII, 4FL2-R406, 4FL2-P505-15.

Further evidence is shown in Figure 6, where the high variability of the conformations of Gleevec and Syk II, both alone or in combination, is evident from the peaks, as compared to P505-15 and R406. Gleevec shows significant variation of the structure disposition, with a value range of 1.5 to 3.8 Å.

Based on these data, we could establish the potential mechanism of the interactions between the competitive ATP inhibitors and the binding site of the protein, which is involved in the inhibition process.

It should be noted that the autophosphorylation site Tyr 525 interacts only with Gleevec, which could explain the different inhibition levels observed in in vitro (IC_50_ 3.81 µM) and in silico (Ki 13.65 nM) studies. Furthermore, the data demonstrated that most of the amino acid interactions established by Gleevec could be relevant in providing better chemical stability and lower binding energy in silico, although this ligand is not specific to Syk. In this paper, we also report that all Syk inhibitors interact with Met450, Leu453, and Pro455; these amino acids are of great importance because of their high levels of Syk specificity [41,42,43].

We postulate that all of the discrepancies between the different methodologies used in this study could be due to numerous biological variables that occur in cultures of infected RBCs (i.e., RBC membrane transport, infection by parasites, ATP consumption) and to the difficulties founded in in silico studies caused by the inability to use fixed concentrations for the tested compounds.

A good correlation was found for the IC_50_ values among in vitro and in silico experiments; R406 and P505-15 followed the same trend and were more effective. Gleevec demonstrated major interactions and had the lowest Ki value in the docking analysis. The free binding energy values confirmed the high affinity of Syk inhibitors toward the catalytic site. The computed energy gap between the HOMO and LUMO was used to determine the chemical stability and molecular features of all tested compounds. All of this information will be useful in facilitating the selection of similar Syk inhibitors and antimalarial compounds. Based on these conclusions and the fact that the parasite cannot mutate an erythrocyte tyrosine kinase, we can speculate that Syk inhibitors could contribute to the potency levels of ACTs. It must also be noted that none of the currently used antimalaria drugs prevent the rupture of infected erythrocytes and reinvasion or inhibit host targets that cannot be mutated by the parasite in order to develop drug resistance. 

Future computational and proteomic analyses will be necessary to better understand the importance of some amino acids in the pattern of interaction, basing the research of new compounds on the catalytic site features in order to improve their in vitro efficacy.

## 3. Materials and Methods 

In this work, we performed a series of in vitro experiments in infected RBCs in order to investigate the biological activity of different Syk inhibitors on *P. falciparum* cultures and evaluate their IC_50_ concentrations. We measured their activity at varying concentrations, for various durations, and at different parasite stages. Furthermore, *in proteomics* studies, the levels of Tyr phosphorylation in oxidized RBCs were quantified using diamide (a reagent that oxidizes sulphydryl groups to the disulfide form). These analyses were followed by the identification of amino acid interactions in the catalytic site of Syk protein through in silico studies.

### 3.1. In Vitro Experiments 

Freshly drawn blood (R+) samples from healthy adults were used to sustain the parasites in in vitro cultures. Healthy adults provided written, informed consent in ASL.1-Sassari. The in vitro studies were conducted using *P. falciparum* (Palo Alto strain), as previously reported for the Palo Alto (PA) strain (mycoplasma-free) according to standard protocols [44,45]. The Palo Alto (PA) strain is a reference parasite strain that is used to study various antimalarial drugs in *P. falciparum*. The PA strain was isolated from a Ugandan patient and is considered a reference strain due to its high genetic stability [46].

Parasite cultures were synchronized as described by Lambros and Vanderberg [47]. Throughout this procedure, *P. falciparum* cultures maintained synchronicity for 2–3 cycles. For all experiments, mature parasites (shizonts and segmenters) after Percoll separation [48] were added to washed RBCs; 12 h after the infection (occurring within 6 h), the cultures were ready for the experimental procedures.

All experiments were carried out by starting with 2% hematocrit and 2% parasitemia. Each well of a 24-multiwell plate contained 500 μl of growth medium treated with different concentrations of the drugs (0.2, 0.8, 1, 2, 4, 8, 10 μM) R406, Syk II, Gleevec, and P505-15 for 24 and 48 h. The parasitemia was evaluated by optic microscopy and the IC_50_ value of each compound was calculated using ICEstimator 1.2 [49,50].

### 3.2. Treatment of Red Blood Cells 

Venous blood was drawn from healthy volunteers following informed consent and pelleted at 1000 g for 10 min at room temperature. After removal of the buffy coat, RBCs were again pelleted and washed 3 times with phosphate-buffered saline (127 mM NaCl, 2.7 mM KCl, 8.1 mM Na2HPO4, 1.5 mM KH2PO4, 20 mM HEPES, 1 mM MgCl2, and pH 7.4) in 5 mM glucose (PBS glucose) to obtain packed cells. RBCs were suspended at a hematocrit level of 30% in PBS glucose and pretreated in different experiments with Syk inhibitor II (Merck, Darmstadt, Germany.), R406 (Selleckchem, Darmstadt, Germany), and Gleevec at different concentrations (0.2, 0.8, 1, 2, 4, 8, 10 μM) for 1 h at 37 °C in the dark, then in the presence of the oxidant diamide at a 2 mM concentration for 45 min. For all the protocols described, untreated controls and controls treated with only 2 mM of diamide were identically processed. To prevent further phosphorylation of band 3, after incubation we washed the cells with cold buffer and the membranes were immediately prepared.

### 3.3. RBC Membrane Preparation

Membrane proteins were prepared at 4 °C on ice as previously described [16]. Briefly, 150 μL of packed RBCs was diluted into 1.5 mL of cold hemolysis buffer (HB) (5 mM disodium phosphate, 1 mM EDTA, pH 8), containing a protease and a phosphatase inhibitor cocktail, then washed up to 4 more times in the same buffer (until membranes became white) in a refrigerated Eppendorf microfuge at  25,000× *g*. The samples were stored frozen at −20 °C until use. The membrane protein content was quantified using the CD Protein Assay (Bio-Rad).

### 3.4. SDS-PAGE

To perform one-dimensional electrophoresis, membrane proteins were solubilized in Laemmli buffer [51] at a volume ratio of 1:1.30 µg of protein-to-antiphosphotyrosine. Then, the samples were put in a thermomixer at 1400 rpm and 28 °C for 30 min. Next, the samples were stored at 95 °C for 5 min, then separated on 8% polyacrylamide gel under reducing and non-reducing conditions. The electrophoretic run was performed on the Bio-Rad Mini-Protean 3 setup. 

### 3.5. Western Blot Analysis 

Proteins separated by SDS-PAGE were transferred to nitrocellulose membranes as previously described with Trans-Blot Turbo Bio-Rad and then probed with antiphosphotyrosine antibody (sc7020, Santa Cruz, CA, USA). This was produced in mice in Santa Cruz, CA, USA, and was diluted to 1:2000. Secondary antibodies conjugated with infrared fluorescent dyes excitable at 680 nm or 800 nm (IRDye, Antimouse 800 CW 926-32210, Li-COR, Lincoln, NE, USA) were then used to visualize the desired antigens with a laser scanner (Odyssey, Licor, Lincoln, NE, USA). Quantitative densitometry analyses of tyrosine phosphorylation levels were carried out by analyzing Western blot images using Image J software. The values were expressed as arbitrary units. The rate of band 3 phosphorylation was expressed as the PTP activity and as a percentage of the maximal activity in RBCs treated with 2 mM diamide. All graphs precisely show the relative phosphorylation, expressed as a percentage of the maximum observed in each experiment (100%). The results show the average of four experiments, normalized to total beta-actin levels. The error bars represent the standard deviations (SDs) of the data. The IC_50_ values of different drugs were calculated using ICEstimator 1.2 software.

### 3.6. Molecular Mechanics (MM) and Quantum Chemicals (QC)

Computational modelling was performed on IBM Blade Center HS22 7870 multiprocessor machines, using OS Ubuntu 16.04 or Windows 10. The small molecules were constructed with standard bond lengths and angles from the fragment database with MacroModel 5.5 [52]. Minimization of structures by conformational search was performed with the MacroModel/BachMin 6.0 program using the AMBER force field.

An extensive conformational search was further carried out using Monte Carlo energy minimization [53] (Ei-E min < 5 Kcal/mole, the energy difference between the generated conformation and the current minimum).

The atomic charges were assigned using the Gasteiger–Marsili method [54]. Representative minimum energy conformations of each compound were optimized using the quantum chemistry program Gaussian 09W with the DFT B3LYP/6-311G method basis set. Visual quantum chemical calculation analysis was performed with GaussView version 5.0 [55,56].

### 3.7. Molecular Electrostatic Potential (MEP)

The molecular electrostatic potentials (MEP) related to the dipole moment, electronegativity, and partial charges, and showing the reactivity of a molecule were computed. Positive potential values reflect nucleus predominance, while negative values represent rearrangements of electronic charges and lone pairs of electrons.

The analyzed MEP were expressed as different colors depending on the densities of organic molecules and electrophilic electrons, with red representing a negative charge and blue representing a positive charge.

### 3.8. Molecular Docking 

All docking tests were performed by considering a 60 × 60 × 60 grid and adopting the default grid spacing (0.375 Å), treating the docking active site as rigid and the ligands as flexible, i.e., all non-ring torsions were considered active (free to rotate).

Binding of the compounds was analyzed using MGLTools 1.5.7rc1 [57] and AutoDock 4.2 docking programs [58,59].

From the estimated free energy values of ligand binding (E.F.E.B., ΔG), the inhibition constant (Ki) for each ligand was evaluated. Ki was calculated using the equation: Ki = exp ((ΔG×1000)/(R×T)), where ΔG is the docking energy, R (gas constant) is 1.98719 cal K−1 mol−1, and T (temperature) is 298.15 K. The protein target Syk complex with the AMP-PNP ligand (PDB ID: 4FL2; resolution of 2.19 Å) was chosen, which is deposited in RCSB Protein Data Bank [60]. The structure was the most defined and complete, except for the first part of the N-terminus (a.a. 1–8) and the interdomain linker region (a.a. 265–336). The crystallographic water molecules were stripped and hydrogen atoms were added using the AutoDockTools (ADT) module.

### 3.9. Molecular Dynamics (MD)

Molecular dynamics (MD) calculations were performed to simulate the interactions with the active site of Syk protein, with the best conformation scores predicted by Autodock for all ligands. The MD protocol) for the production simulations were carried out using the Particle Mesh Ewald Molecular Dynamics (PMEMD) version included in the AMBER14 program [61], after careful relaxation of the system using minimization and equilibration protocols.

10 nanoseconds of molecular dynamics production trajectory was saved (5000 frames of 0.002 nanoseconds). The ionizable residues were set to their normal ionization states at pH 7, while the protein atoms and all water molecules of the crystal structure were surrounded by a periodic box of TIP3P32 water molecules that extended 10 Å from the protein. Counterions (Cl^−^ or Na^+^) were placed by xleap to neutralize the system with a Ewald force field and TIP3P water [62].

The ff10 version of the AMBER force field was used to model the protein, and the General AMBER Force Field (GAFF) was used for the organic ligand using the Austin Model 1–Bond Charge Corrections (AM1-BCC) partial charges derived from the antechamber program of the AMBER suite. In the MD simulation protocol, the SHAKE algorithm was used to constrain all bonds involving hydrogen atoms. A non-bonded cutoff of 8.0 Å was used. Langevin dynamics were used to control the temperature (300 K) using a collision frequency of 1.0 ps-1, along with isotropic position scaling to maintain the pressure (1 atm). Periodic boundary conditions were applied to simulate a continuous system. To include the contributions of long-range interactions, the particle mesh Ewald (PME) method was used with a grid spacing of 1 Å combined with a fourth-order B-spline interpolation to compute the potentials and forces in between grid points. The trajectories were analyzed using the Processing Trajectory program (PTRAJ) module of AMBER.

Molecular mechanics and Poisson–Boltzmann (or generalized Born) surface area (MM/PB(GB)SA) calculations and analyses were done with the MM-PBSA program in Amber14 suite.

Graphical representation of the hypothetical positions derived from the docking calculations and the trajectory analysis of molecular dynamics calculations was performed using Chimera [63] and Visual Molecular Dynamics (VMD) [64] software.

Calculations and energy comparisons were conducted using the method proposed by Ross Walker [65].

### 3.10. Validation of Molecular Docking Protocol

**Figure 7:**
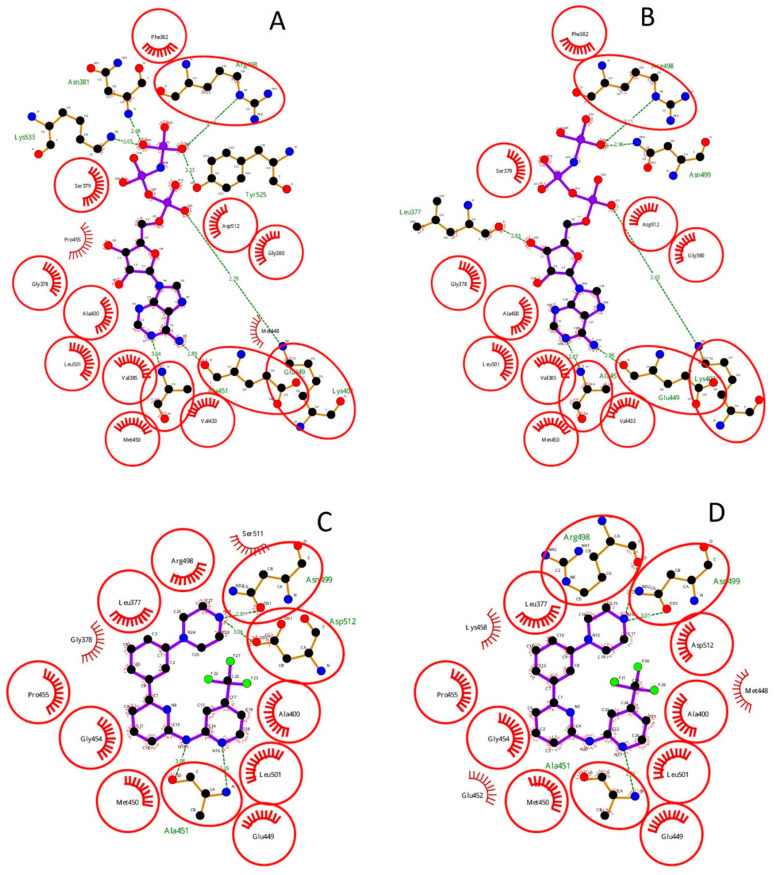
Two different approaches used to validate the docking analysis protocol related to X-ray images of 4FL2 (**A**) and 4F4P (**C**) crystals. The first test consisted of re-docking among 4FL2 and its ligand ANP (**B**), while the second test involved the docking of 4FL2 with the ligand 0SB (**D**). The 2D diagrams were achieved through the LigPlot+ tool.

The reliability of the docking approach was further verified with two methods, with one test performed by extracting the phosphoaminophosphonic acid adenylate ester (ANP) from the catalytic site of 4FL2, with a 2.19 Å resolution crystal structure, leading the re-docking. After repositioning the ANP into the protein, the new ANP location was the same as in the original X-ray structure, with RMSD 1.54 Å, which was repositioned with only minimal conformational changes, hence confirming the reliability of the system (RMSD 1.54 Å). The second test was conducted by extraction of the N-{6-[3-(piperazin-1-yl)phenyl]pyridin-2-yl}-4-(trifluoromethyl)pyridin-2-amine (0SB) from X-ray images of 4F4P.pdb, with a 2.37 Å resolution, which was docked with the same macromolecule derived from 4FL2.pdb. After the docking, the new 0SB location was the same as in the original X-ray structure, with RMSD 0.99 Å, which was repositioned with only minimal conformational changes, hence further confirming the reliability of the system (Figure 7). The multiple ligand–protein interactions were analyzed through a 2D diagram in LigPlot+ [66] and the RMSD values of 1.54 Å and 0.99 Å were consequently evaluated in ANP and 0SB experiments, respectively. We could assume the validation of the docking protocol to be satisfactory, considering the values obtained were lower than 2 Å.

## 4. Conclusions 

Molecular docking is one of the most widely used computational approaches in the design of structure-based drugs [67,68]. This approach can be used both to identify the correct conformation of the ligand within the target binding pocket and to estimate the interaction energy between a target and a ligand [69,70]. In the present study, different Syk inhibitors were investigated using different methodologies to test their efficacy in vitro and in silico as promising new antimalarial drugs to block Syk phosphorylation events in RBCs infected by parasites. Thorough the computational studies, we gathered useful information about the tested inhibitors, analyzing their chemical features and the patterns of interaction in the Syk protein pocket, proving their inhibitory activity in in vitro experiments. Based on the above data and other publications from our labs, we demonstrated that these compounds actually block the phosphorylation of Tyr residues in protein band 3. The in vitro experiments using a proteomic approach allowed us to notice that Syk inhibitors terminate the life cycle during the process of merozoite egress from the infected RBC, leading to a decrease of band 3 phosphorylation and avoiding the destabilization and weakening of the erythrocyte membrane. The biological data obtained by evaluating the IC_50_ values using Western blotting for all tested compounds confirmed the results achieved in in silico studies. The development of new drugs that can effectively kill malaria parasites is of increasing importance as the threat of drug resistance continues to grow. However, as highlighted above, the findings of this study require further exploration and testing to elucidate or confirm the role of Syk inhibitors as potential drug targets.

## Figures and Tables

**Table 1:** IC_50_ values obtained from the densitometry analysis performed on anti-p-Tyr Western blotting membranes.

Syk Inhibitors	IC50 (μΜ)
**P505-15**	0.64
**Gleevec**	0.77
**R406**	0.83
**Syk II**	1.72

**Table 2:** Approximate IC_50_ values for each drug were determined by treating ring-stage cultures of *P. falciparum*, Palo Alto strain, with different concentrations of each drug and by quantitating residual parasitemia 24 and 48 h later.

Palo Alto Strain	24 h		48 h	
	IC50 (μΜ)	Range (μΜ)	IC50 (μΜ)	Range (μΜ)
P505-15	0.83±0.06	0.78−0.90	0.49±0.07	0.42−0.61
R406	2.62±0.83	1.42−3.85	0.55±0.19	0.25−0.92
Gleevec	3.81±0.55	3.24−4.95	1.55±0.13	1.32−1.74
SYK II	5.01±0.44	4.65−5.87	0.90±0.16	0.82−1.25

**Table 3:** The data shown represent the H-bond binding found in docking analyses from the different inhibitors (Gleevec, R406, Syk II, and P505-15) interacting with Syk protein. Abbreviations: hydrogen donor, HD; oxygen acceptor, OA; nitrogen acceptor, NA; Debie, D; and Angstrom, Å. The cross-bridge H-bond interactions with the same aa are listed in bold”.

H-Bond Interaction								
Ligands	Pose	%	H-Bond	Ligands Atom	Protein Atom	Distance (Å)	LogP	D. M. (Debye)
Gleevec	1	73	4	N33(NA)	Lys402:HZ1(HD)	2.217	3.83	5.1101
				O10(OA)	Ala451:HN(HD)	2.393		
				N38(NA)	Arg498:HE(HD)	2.147		
				H27(HD)	Asp512:OD2(OA)	1.995		
R406	11	16	3	H9(HD)	Leu377:O(OA)	1.875	3.37	3.4870
				H25(HD)	Ala451:O(OA)	**2.262**		
				O36(OA)	Ala451:HN(HD)	**2.317**		
SykII	1	56	7	H23(HD)	Glu449:O(OA)	**2.075**	1.22	4.4175
				H24(HD)	Glu449:O(OA)	**2.233**		
				O25(OA)	Ala451:HN(HD)	2.299		
				H29(HD)	Arg498:O(OA)	2.063		
				H30(HD)	Ser511:HG(HD)	2.558		
				H8(HD)	Asp512:OD2(OA)	**2.191**		
				H30(HD)	Asp512:OD2(OA)	**2.025**		
P505-15	8	37	4	H8(HD)	Ala451:O(OA)	**2.301**	1.02	2.4285
				O21(OA)	Ala451:HN(HD)	**1.888**		
				H23(HD)	Glu449:O(OA)	**1.940**		
				H24(HD)	Glu449:O(OA)	**2.448**		

**Table 4:** Hydrophobic interactions and H-bonds between Syk inhibitors and proteins. The bonds are shown highlighted with * (1 H-bond) and with ** (2 H-bonds). Amino acids that are underlined have a high level of Syk specificity. The amino acid Tyr525 is double underlined, which is an autophosphorylation site of Syk protein that forms an interesting interaction with Gleevec.

R406	Syk II	Gleevec	P505-15
Leu377 *	Leu377	Arg338	Leu377
Gly378	Gly378	Leu377	Val385
Ser379	Val385	Ser379	Ala400
Gly380	Ala400	Gly380	Met448
Val385	Lys402	Asn381	Glu449 **
Ala400	Met448	Phe382	Met450
Lys402	Glu449 **	Val385	Ala451 **
Val433	Met450	Ala400	Glu452
Met448	Ala451 *	Lys402 *	Gly454
Glu449	Gly454	Met448	Pro455
Met450	Pro455	Met450	Lys458
Ala451 **	Lys458	Ala451 *	Leu501
Glu452	Arg498 *	Leu453	
Gly454	Leu501	Gly454	
Pro455	Ser511 *	Arg498 *	
Lys458	Asp512 **	Asn499	
Leu501		Leu501	
Asp512		Ser511	
		Asp512 *	
		Tyr525	
		His531	

**Table 5:** Data showing the binding energy and inhibition constant (Ki) values obtained from the docking analysis of Syk inhibitors with 4FL2. Abbreviations: mean binding energy, M.B.E; estimated free energy of binding, E.F.E.B; estimated inhibition constant, E.I.C.

Ligand	M.B.E	E.F.E.B	E.I.C, Ki
**Gleevec**	−10.57	−10.73	13.65 nM
**P505-15**	−7.69	−8.81	346.88 nM
**R406**	−7.28	−7.94	1.50 μM
**SykII**	−6.80	−6.95	8.03 μM

## Abbreviations

ACTs	Artemisinin-based combination therapies
Syk	Spleen tyrosine kinase
ANP	Phosphoaminophosphonic acid adenylate ester
OSB	N-{6-[3-(piperazin-1-yl)phenyl]pyridin-2-yl}-4-(trifluoromethyl)pyridin-2-amine
MD	Molecular dynamics
QM	Quantum mechanics
QC	Quantum chemistry
RMSD	Root mean square deviation
RBCs	Red blood cells
HOMO	Highest occupied molecular orbital
LUMO	Lowest unoccupied molecular orbital
MEP	Molecular electrostatic potential
